# Being in the Know: The Role of Awareness and Retrieval of Transient Stimulus-Response Bindings in Selective Contingency Learning

**DOI:** 10.5334/joc.227

**Published:** 2022-06-09

**Authors:** Mrudula Arunkumar, Klaus Rothermund, Wilfried Kunde, Carina G. Giesen

**Affiliations:** 1 Department of General Psychology II, Friedrich Schiller University Jena, DE; 2 Experimental and Cognitive Psychology, Julius-Maximillians-University Würzburg, DE

**Keywords:** contingency learning, stimulus-response binding, episodic retrieval, saliency

## Abstract

Previous studies demonstrated that contingency learning can be both (a) unaware (Schmidt et al., 2007), and (b) explained in terms of an automatic retrieval of stimulus-response bindings from the last episode in which the cue stimulus has been presented (Giesen et al., 2020; Schmidt et al., 2020). We investigated whether learning is selective in a contingency learning paradigm in which pairs of salient and nonsalient cues that were equally predictive of responses to targets (digits) were presented simultaneously. In two pre-registered experiments (total N = 137), we found stronger contingency learning for salient compared to non-salient cues. Transient stimulus-response binding and retrieval processes did not contribute to these selective learning effects in contingency learning, which were instead driven by contingency awareness. Our findings indicate that under conditions of high saliency, contingency learning is mediated by conscious rule detection for which retrieval of transient stimulus-response bindings is irrelevant.

In visual perception, stimuli that deviate from their immediate surroundings are more likely to catch our attention than stimuli that are similar to the contexts in which they are presented. Be it the proverbial ‘woman in a red dress’ in a street full of business commuters, an adult in a group of three-year-old children, or a Caucasian European in a Buddhist monastery – what all of these examples have in common is that one visual exemplar will stand out from all other exemplars due to a dominant feature that distinguishes this exemplar from all others, rendering it more salient (Duncan & Humphreys, 1989). Being more attention-grabbing equips the exemplar with an advantage in a cascade of cognitive processes that typically follow, as for instance, access to memory, retention, or learning.

In this article, we investigate how differences in relative saliency of simultaneously presented stimuli affect learning for these salient and nonsalient visual stimuli. This research was motivated by evidence for selective learning in the field of Pavlovian Conditioning (for an overview, see Dickinson & Macintosh, 1978), which show little or no learning for a conditioned stimulus (CS, e.g., a light) when other CS predict the occurrence of an unconditioned stimulus (US, e.g., food). For instance, in overshadowing, reliable conditioning for a CS occurs if that CS is presented in isolation but not if the CS is presented simultaneously with another, more salient CS (Pavlov, 1927; Kamin, 1968; Kehoe, 1982; Mackintosh, 1976; Miles & Jenkins, 1973; Wasserman & Miller, 1997).

Evidence for selective learning is not only based on animal studies but also on findings from contingency learning (CL) in humans (e.g., Schmidt & De Houwer, 2019; for an overview, see De Houwer & Beckers, 2002). There seems to be a preference of learning for one cue over the other when cues are redundantly predictive of the response or differ in relative saliency (McLaren et al., 2014; Endo & Takeda, 2004), although these studies did not test cues in isolation. Similarly, Schmidt and De Houwer (2019) did not experimentally manipulate the saliency of competing cues but rather studied mutual overshadowing of different stimulus features (shapes and word meaning) of a compound cue, which might have differed not only with regard to their saliency but also in other respects (e.g., semantic vs. non-semantic processing).

Against this background, we explored the phenomenon of selective learning in a contingency learning paradigm more systematically, for several reasons.^1^ First, we tested whether there is evidence for cue preference in contingency learning in an experimental setup with two competing cues that differ only with regard to their saliency. Second, we intended to find out more about the underlying mechanisms that produce these selective contingency learning effects as well as additional outcomes of these mechanisms. In detail, we aimed to clarify the role of automatic retrieval of incidental stimulus-response bindings in producing selective learning effects for salient versus nonsalient cues. The second research aim is motivated by recent findings which support the view that contingency learning effects can be explained by an automatic retrieval of stimulus-response bindings (SRB) from memory (Giesen et al., 2020; Schmidt et al., 2020; see also Jiménez et al., 2021; Rothermund et al., 2022). SRBs reflect links between a presented stimulus and an executed response, which are stored in episodic memory (*event files*; Hommel, 1998). The next time the same stimulus is presented, this will retrieve the bound response, thus resulting in faster performance of the same response and impeded performance whenever a different response is required. Notably, SRBs are not restricted to task-relevant stimuli; instead, other task-irrelevant information like distractors that are simultaneously presented can be linked to responses and retrieve them upon later repetition (Rothermund et al., 2005; Frings et al., 2007). Retrieval of SRBs is a robust phenomenon that applies to many stimuli, responses, and modalities (for overviews, see Frings et al., 2020; Henson et al., 2014). However, there is an ongoing debate about whether SRBs are only stored transiently (as a mere by- product of distributed processing, cf. Hommel, 1998; Hommel et al., 2001) or whether they also serve as a basis for longer lasting learning phenomena (*unitary view*, e.g., Schmidt et al., 2016, 2020). Research findings on this issue are still scarce and mixed (e.g., Colzato et al., 2006; Herwig & Waszak, 2012; Schreckenbach & Rothermund, 2022; for a detailed discussion, see Moeller & Frings, 2014). Recently however, Giesen and colleagues (2020; see also Schmidt et al., 2020) could show that long term contingency learning effects can be explained by episodic retrieval of SRBs from memory. Their findings suggest a close parallel between binding and long-term learning phenomena, which is consistent with the idea that SRB effects as well as contingency learning effects can result from the very same memory storage and retrieval processes (see Schmidt et al., 2016).

Another close parallel between SRB and contingency learning effects concerns the role of awareness: In contingency learning paradigms like the color-word contingency task (e.g., Schmidt et al., 2007), but also in SRB tasks that included contingency manipulations (Giesen & Rothermund, 2015), participants typically pick up the contingencies between stimuli and responses in their behavior without explicit instructions, and without necessarily becoming aware of the contingencies or being influenced by the awareness of these contingencies (Schmidt et al., 2007; Giesen & Rothermund, 2015). This suggests that awareness (i.e., being able to verbally report stimulus-response contingencies in a propositional form) does not seem to be crucial for some types of contingency learning or SRBs. However, Schmidt and De Houwer (2019) only obtained evidence for selective contingency learning (here: overshadowing) when participants were explicitly informed about contingencies but found no overshadowing effects under conditions of incidental learning. Thus, we aimed to more closely investigate (a) the role of automatic retrieval of SRBs in producing selective contingency learning effects as a function of relative cue saliency and also (b) whether these effects are mediated by contingency awareness.

To investigate these research questions, we designed the following task: Participants performed a speeded categorization task and classified centrally presented digits (targets) as odd or even via key press. Target displays were preceded by a compound cue display, which consisted of a circle of eight letters (see Figure 1A; Figure 2). Letters were irrelevant for the digit classification task, and thus can be considered as distractors. Each compound display consisted of eight letters, two of which were *equally predictive* of the upcoming classification response (90% valid; 10% invalid) and thus served as *cues*, whereas the remaining six letters were non-predictive. Pairs of predictive cues were always presented together during the learning phase. Importantly, the two cues of a pair differed in saliency, meaning that one cue was always presented in red whereas the other letters were presented in blue color, which rendered the cue salient (Theeuwes, 1992; 2004), whereas the other cue of the pair was presented in the same color as the remaining, non-predictive distractors (blue), which rendered it nonsalient. Saliency of cues was experimentally manipulated for a given set of compound cue trials, referred to as *learning displays*.

**Figure 1:**
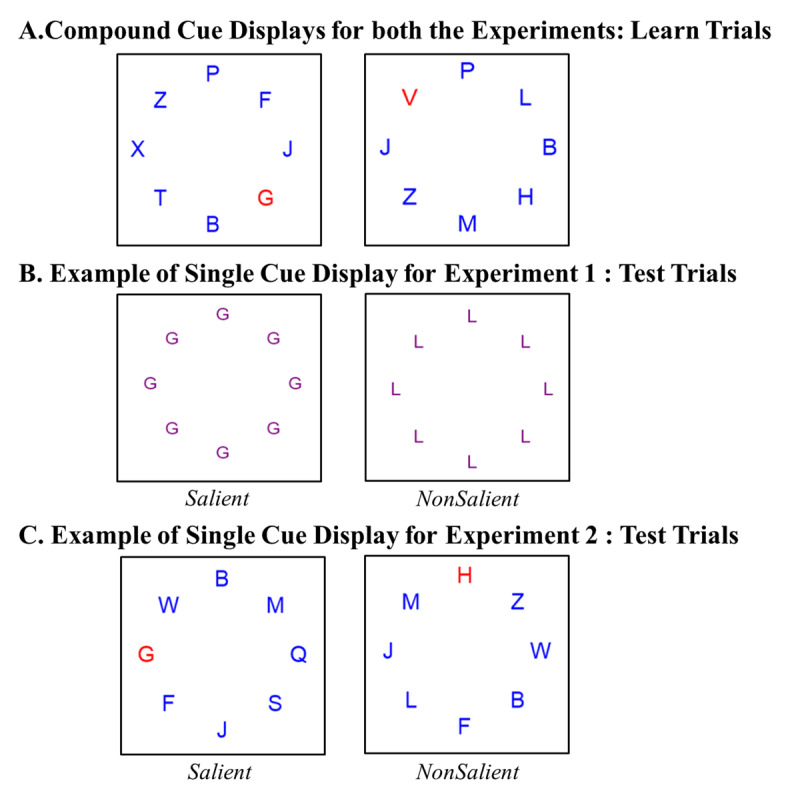
**A.** Examples of compound cue display containing G (salient) and X (nonsalient) letters (left compound cue display) and V (salient) and L (nonsalient) letters (right compound cue display). **B.** Examples of single cue display in Experiment 1 containing only the salient or the nonsalient cue presented in a neutral colour purple. **C.** Examples of single cue display of Experiment 2, presenting either the salient (red G and random blue letters) or nonsalient (blue L but a random red letter) single cue.

**Figure 2:**
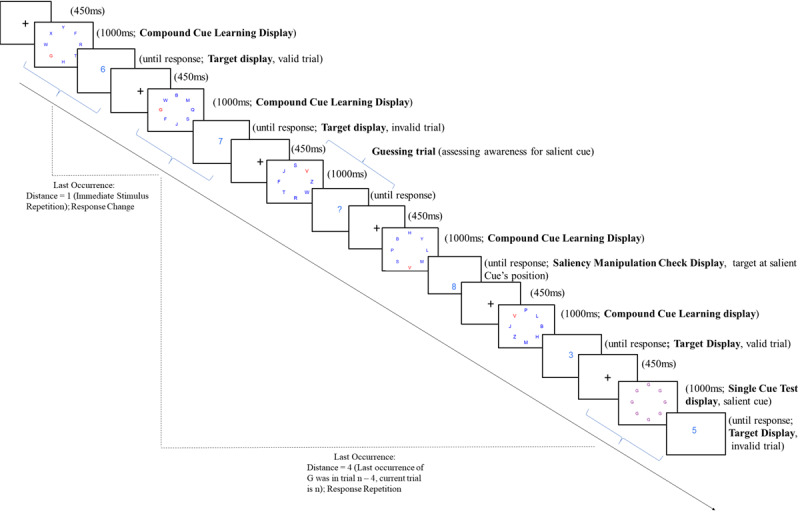
Example flow of Experiment 1 containing each of the display types, stimuli are not drawn to scale. It is important to note that in the actual flow of the experiment the trials are presented randomly within an intermixture of single and compound cue displays.

In a later test phase, contingency learning effects were assessed by presenting single cue *test displays* (see Figure 1B, C; Figure 2), wherein either the previously salient or nonsalient cue was presented separately without the other co-predictive cue. This was in the form of a circle of eight identical letters of either the identity of salient or nonsalient letter (Experiment 1) or with a display similar to the learning displays in which only the salient *or* the nonsalient letter was presented among seven other letters in a circle (Experiment 2). These single cue displays were always presented before the digit occurred (Figure 2). In Experiment 1, during the test phase, cue displays were no longer predictive of digit categorization responses, that is, we had an equal number of valid and invalid trials, in which the digit corresponded to the category that had been presented more (less) frequently after the cue during the learning phase. However, in Experiment 2, the contingency ratio of 90:10 was maintained for the test displays. Contingency learning effects were computed during the test phase as the performance difference between valid and invalid trials. Contingency learning effects were estimated separately for each cue, which allowed us to assess the effect of saliency on learning, which is reflected in larger contingency learning effects for salient compared to non-salient cues during the test phase.

## Study aims and hypotheses

Our first research aim was to demonstrate selective learning in an experimental setup using a contingency learning paradigm with two competing cues that differ in saliency. Selective learning would be evidenced by stronger contingency learning for salient than for nonsalient cues (statistically, this is reflected in a test trial validity × cue saliency interaction). Second, we aimed to study the role of contingency awareness in selective learning for task irrelevant cues. This was done by assessing whether contingency awareness (collected in on-task and off-task measures, see Figure 2) moderated selective learning effects. Third, we assessed the role of retrieval of incidental SRBs in producing these learning effects. This was done by employing hierarchical multilevel analyses which test whether the selective learning effects for salient cues would remain significant after statistically controlling for the retrieval of SRB’s (Giesen et al., 2020).

## Experiment 1

Exact method, hypotheses, exclusion criteria, and planned analyses of Experiment 1 were preregistered on OSF with the AsPredicted.com template (10.17605/OSF.IO/SWX5E).

### Method

#### Participants

Seventy participants (N = 70, *M_age_* = 23.7) were recruited through the online platform Prolific Academic (https://www.prolific.co/). An *a priori* power analysis (G*Power 3; Faul, et al., 2007) indicated that we need 70 participants in order to detect a small to medium sized effect of d_z_ = 0.4 (Brysbaert, 2019) with a power of 1–β = .90. We pre-screened participants and only sampled participants with English as first language, aged between 18–35 years, and with an approval rate of 65% or higher. The experiment lasted 20–25 minutes and the participants were compensated according to the norms of Prolific (2.70£). Participants also had an opportunity to gain a bonus of 60 pence based on their performance in the guessing trials. Participants with error rates higher than 15% were excluded from the analysis (N = 1) and were replaced by new participants. Informed consent was given by pressing the “j” key upon reading the consent form which was displayed at the start of the study. Ethical approval was not required since no cover-story or otherwise misleading or suggestive information was conveyed to participants (this procedure is in accordance with the ethical standards at the Institute of Psychology of the FSU Jena).

#### Apparatus and stimuli

The Experiment was programmed in Psychopy3 (v2020.1.3 Pierce et al., 2019) and hosted on Pavlovia (https://pavlovia.org/) to enable online data collection. Participants were instructed to open it only on a laptop (13” to 15” laptop). The stimuli consisted of letters that were task irrelevant (G and V served as salient cues; X and L served as nonsalient cues; the remaining distractor letters were randomly sampled from the alphabet) and digits (1 to 9) which were task relevant. Stimuli were presented in Arial font, with the letter height of 0.04 according to the units in PsychoPy. On compound cue learning displays, salient cues were presented in red, nonsalient cues and non-predictive distractor letters were presented in blue. Positions of salient and nonsalient cues were chosen randomly for each display with the restrictions that the nonsalient and salient cue would never appear adjacent to one another or directly opposite. This eliminates three positions (two on either side of the salient cue and one directly opposite); thus, the nonsalient cue appeared randomly at one of the remaining four locations. In single cue test displays, all letters were presented in purple. In target displays, the number was presented in blue on a white background. The task was to judge whether the target number was odd or even via left/right key press (odd: press “e”; even: press “u”).

#### Procedure

Instructions were displayed on the screen at the start of the experiment. Participants were told that a circular display of letters will be presented in a first display, followed by second display in which a number will be shown in the center of the screen. Their task was to simply look at the number and press “e” if the number is odd and “u” if the number is even. Participants were also briefly told that on some trials, a “?” will be presented instead of a digit in the second display. In these situations, participants were instructed to press the response key that corresponded to the number category they expected to occur. These trials were used to measure their level of contingency awareness (see explanation for “guessing trials” below).

A short attention check with four questions was presented to make sure that the participants read the instructions well. Once the attention check was passed successfully, the participants moved on to the practice block containing 16 trials (8 compound cue learning displays and 8 single cue test displays). Feedback was given for incorrect or too slow (>2s) responses. The practice block was repeated if participants produced 50% or more wrong responses. Upon successful completion of the practice block, the experiment began (see Table 1 for the flow of the experiment). First, participants worked through 40 valid compound cue learning trials, giving them a *headstart* to establish the contingencies (Schmidt et al., 2010). Immediately following these headstart trials, 80 compound cue learning displays were presented (72 valid, 8 invalid) during the learning phase, along with 16 contingency guessing displays that were presented in between to assess contingency awareness. After a self-paced break, the experiment continued with a random intermixture of 200 compound cue learning displays (180 valid, 20 invalid), which were presented to avoid extinction effects, 80 single cue test displays (consisting of 40 salient single cue displays with 20 valid and 20 invalid trials, and 40 nonsalient single cue displays with 20 valid and 20 invalid trials). In addition to the learning displays, there were 16 trials that had a compound cue display followed by a target display wherein the target number did not appear in the center but rather appeared at the position of the salient cue (8 trials) or nonsalient cue (8 trials). These trials were added to check whether the saliency manipulation worked in our design (see explanation for “manipulation check display trials” below). These 16 saliency manipulation check displays were also intermixed among the other type of trials mentioned above. Another self-paced break was given after 148 trials. The experiment ended with four single cue test displays that were followed by a guessing display and six questions to assess subjective contingency awareness with a funneled debriefing (see Table 1). Questions 1 and 2 were “*Did you have an impression that an odd/even number appeared after a particular red letter? Press ‘j’ for yes and ‘n’ for no and ‘t’ if you do not know*”. Questions 3 to 6 were “*What response almost always followed a red “V”/red “G”/“L”/“X”? Press the relevant response key (“e” or “u”) on the keyboard or press “t” if you do not know*”.

**Table 1:** Flow of the main experiment in Experiment 1 and Experiment 2.


EXPERIMENT 1			

TRIAL	DISPLAY 1	DISPLAY 2	VALIDITY (VALID/INVALID)

Trial 1–40	Compound Cue learning display	Target display	40/0

Trial 41–136, random mix of:	Compound Cue learning display	Target display	72/8

	Display resembling Compound Cue learning trials with either salient/nonsalient cue	Guessing display	–

Trial 137–432, random mix of:	Compound Cue learning display	Target display	180/20

	Single Cue test display	Target display	40/40

	Compound Cue learning display	Saliency Manipulation check display	16/0

Trial 433–436	Display resembling Single Cue Test displays with either salient/nonsalient cue	Guessing display	–

	Short funnelled questionnaire: 6 questions		

**EXPERIMENT 2**			

Trial 1–40	Compound Cue learning display	Target display	40/0

Trial 41–456, random mix of:	Compound Cue learning display	Target display	180/20

	Single Cue test display	Target display	180/20

	Compound Cue learning display	Saliency Manipulation check display	16/0

Trial 456-464	Display resembling Single Cue Test displays with either salient/nonsalient cue	Guessing display	–

	Short funnelled questionnaire: 6 questions		


*Manipulation of display types*. Different display types were manipulated as follows. *Compound cue learning trials* consisted of a circular arrangement of eight distractor letters, two of these were cues for the upcoming target classification response: Specifically, G and X predicted the “right” key response and were followed by an even number 90% of the time (valid trials) and were followed by an odd number 10% of all times (invalid trials). Similarly, V and L predicted the “left” key response and were followed by an odd number 90% of all times, and were followed by an even number 10% of all times. Saliency was manipulated experimentally by presenting G and V in red (salient cues), whereas X and L were displayed in blue (nonsalient cues) among six other random (non-predictive) letters that were presented in blue (see Figure 1).

*Single cue test displays* resembled the compound cue learning displays but differed in the aspect that (a) all eight letters were identical and displayed either the salient or nonsalient cue and (b) all letters were presented in purple. Importantly, half of all single cue test displays were followed by a digit that matched the more (less) frequent response category for the respective cue, resulting in an equal number of valid and invalid cue displays for the test displays. 50% of these single cue test displays presented cues that were salient in the compound cue learning display (G and V) and the remaining half presented nonsalient cues (L and X) from the compound distractor display (See Figure 1 for examples of the displays).

Two other display types complemented the task in which we modified target displays: First, we interspersed *manipulation check* displays to test the saliency manipulation. In these displays, the target digit appeared at the location of either the salient or nonsalient cue of the preceding compound cue learning display. All the target digits for these saliency manipulation check displays were valid with the contingency. Classification responses should be faster when digits were presented at the position of the salient cue compared with the position of the nonsalient cue. Furthermore, we introduced *guessing displays* to assess contingency awareness. In these displays, no digit appeared. Instead, a question mark was presented centrally. There were two types of manipulation within these *guessing displays* – one, where the guessing display was preceded by a cue display that almost resembled a compound cue learning display with the only difference that either the salient or the nonsalient cue was shown. These guessing displays were interspersed among other compound cue learning displays within the experiment right after the *headstart* learn trials. It provided a form of online measurement of the awareness of contingencies for the predictive nature of (non-)salient cues for upcoming target classification responses during the course of the experiment (see Figure 2). The other type of guessing displays were presented at the last part of the experiment (just before the the funneled debriefing): Four single cue test displays were presented, followed by a guessing display. Participants were instructed that whenever they saw a question mark instead of a number, they should guess and indicate whether they expected an even or odd number via key press (cf. Giesen & Rothermund, 2015). Performance on guessing displays was incentivized by earning additional money: correct responses that confirmed the to-be-learned contingency of the preceding cue trial were rewarded with +3 pence; wrong responses were punished with -3 pence. Positive scores were added to the standard payment; in case of zero or negative scores, participants gained the regular compensation.

Each trial sequence was as follows (see Table 1 and Figure 2): A trial started with a flashing fixation cross (450ms), followed by the first display (compound cue learning display or single cue test display; 1000 ms). Immediately after the first display (ISI = 0 ms), the second display appeared which could be either a target display, a guessing display, or manipulation check display (presented until response). At the bottom of the screen of the second display, the participants received feedback when they pressed an incorrect key (“Error! Press correct key to continue”) or took more than two seconds to respond (“Respond faster! Press correct key to continue”). Responses in guessing trials were followed by performance feedback (trial-wise change and total score) for 2000ms after which the next trial started.

#### Design

The experiment comprised a 2 (single cue test display validity: valid vs. invalid) × 2 (cue saliency in compound learning displays: salient vs. nonsalient) within-subjects design. To test for contingency learning, target display categorization performance (RT; errors) after single cue test displays served as dependent measure.

### Results

#### Data preparation

All participants were included in the analysis. Reaction Time (RT) outliers were computed using Tukey cut-offs (Tukey, 1977). RTs that were more than 1.5 interquartile ranges above the 75th percentile of the individual RT distribution or below 200 ms were exempted from the analysis (5.09%). RTs of incorrect trials were also not used for the analyses (6.41%). All the data files and analyses scripts are openly available via OSF (https://osf.io/sa7eb/).

#### Manipulation checks

We computed two kinds of manipulation checks: First, we analyzed the contingency learning effects for the target displays that followed compound cue learning trials, as a mean RT difference for the more frequent (i.e., valid) vs. less frequent (i.e., invalid) combination of cues and target digits. Participants showed a significant contingency learning effect for RT, *t*(69) = 8.52, *p* < .001, d_z_ = 1.01, with a mean difference of Δ_invalid – valid_ = 51 ms, and errors, *t*(69) = 7.47, *p* < .001, d_z_= 0.89, Δ_invalid – valid_ = 14.11%. This shows that the contingency manipulation worked and learning occurred.

Second, to check whether the saliency manipulation had an effect on participants’ attention, only the performance on the 16 saliency manipulation check trials was analyzed based on the position of the target (position of salient vs. nonsalient cue). Participants performed significantly faster when the target was presented at the location of the salient compared to that of the nonsalient cue, *t*(69) = 2.44, *p* = 0.008, d_z_= 0.29, with a mean difference Δ_nonsalient-salient_ of 17 ms. For error rates, there was no significant difference between performance in the trials where the target was in the salient cue’s position versus the nonsalient cue’s position, *t*(69) = 1.33, *p* = .09, Δ_nonsalient-salient_ = 1.5%. These results show that the saliency manipulation was successful.

#### Transfer effects of contingency learning for salient and nonsalient cues on single cue trials

**Figure 3:**
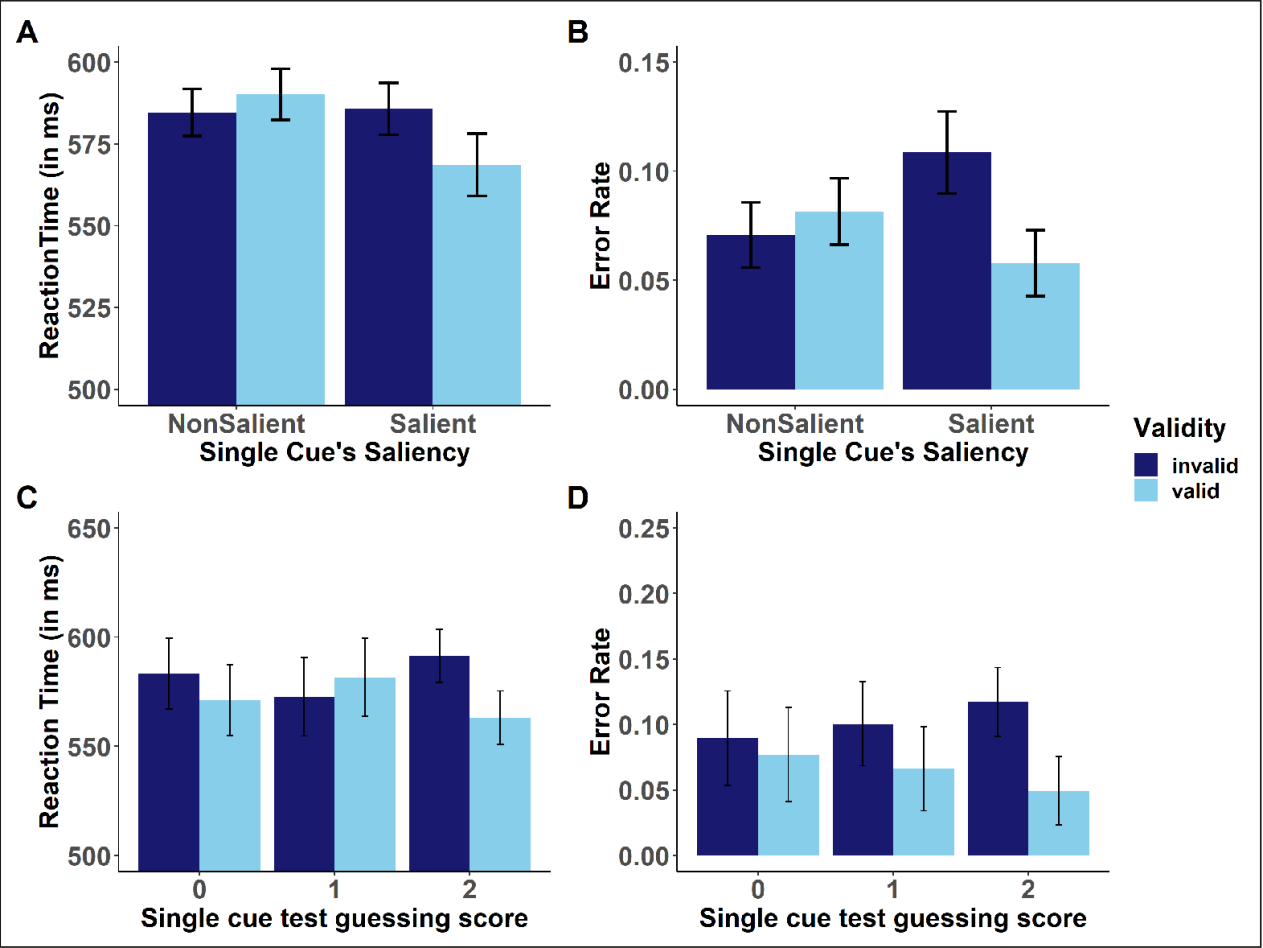
Top Row: Performance on target displays as a function of single cue display validity and cue saliency for mean RT (A) and mean error rates (B). Bottom row: Validity effects for salient cues as a function of participants’ achieved guessing score following single cue test displays for mean RT (C) and mean error rates (D). Error bars represent 95% CI from standard error of each condition as explained in Morey (2008).

To test for transfer effects of contingency learning to the test trials, performance on target displays following the single cue test displays were analyzed. A 2 (Cue Saliency: salient vs nonsalient) × 2 (Validity: valid vs invalid) analysis of variance (ANOVA) with repeated measures was computed on the aggregated mean RT and errors for the factorial design. For RTs, the analysis yielded a main effect of saliency, *F*(1,69) = 5.68, *p* = .019, η_p_^2^ = .08, indicating faster responses after salient than nonsalient single cue displays. This main effect was qualified by a significant interaction between validity and saliency, *F*(1,69) = 8.02, *p* = .006, η_p_^2^ = .10. Follow-up *t*-tests showed that as expected, the contingency learning effect (i.e., the effect of validity) was significant for the salient cues, *t*(69) = 2.71, *p* = .004, Δ_invalid – valid_ = 17 ms, d_z_ = 0.32, but was not significant for nonsalient cues, *t*(69)= –1.11, *p* = 0.864, Δ_invalid – valid_= –5 ms, d_z_ = –0.13 (see Figure 3). No other effect was significant (*F* < 2.5, *p* > .15).

For error rates, the ANOVA yielded a main effect of validity, *F*(1,69) = 5.47, *p* = .022, η_p_^2^ = .07 and a significant interaction between saliency and validity, *F*(1,69) = 13.9, *p* < .001, η_p_^2^ = .17. Follow-up *t*-tests showed that as expected, the contingency learning effect was significant for the salient cues, *t*(69) = 3.95, *p* < .001, d_z_ = 0.47, Δ_invalid – valid_ = 5%, but was not significant for nonsalient cues, *t*(69) = –0.991, *p* = .836, d_z_ = 0.11, Δ_invalid – valid_ = –1% (see Figure 3). No other effect was significant (*F* < 1).

Altogether, these results indicate that there is learning only for the cue that is salient and not for nonsalient cues: Performance on target displays was better (worse) for valid (invalid) cue-target sequences for salient single cue displays than after nonsalient single cue displays. This indicates that contingencies were selectively learnt only for salient cue-response combinations during compound learning trials.

#### Role of contingency awareness

To investigate the role of contingency awareness, we analyzed answers in the various awareness on-task and off-task measures as a function of cue saliency. For all measures, performance was significantly better for salient compared with nonsalient cues (see Table 2), which indicates that participants had awareness for the predictive nature of salient cues. We then were interested whether individual awareness can explain the validity effects obtained for salient cues. For this purpose, we analyzed validity effects (for salient cues only) as a function of participants’ mean accuracy scores in guessing trials for single cue test displays for RT and errors (see Figure 3). Overall, N = 12 had 0 correct answers, N = 16 had a score of 1 correct answer (these participants were regarded as performing below or at chance level); N = 42 had the maximum score of 2 correct answers and thus performed better than chance level. For RT, a 2 (validity) × 3 (single test cue contingency awareness score) MANOVA with two orthogonal contrasts on the last factor (the first contrast compared participants with performance below or at chance level against those who performed better than chance [1 1 –2], the second contrast compared participants who performed below vs. at chance level [–1 1 0]) revealed a significant interaction, *F*(2,67) = 3.16, *p* = .049, η_p_^2^ = .09. Planned contrasts indicated that this interaction was driven by participants who yielded a score of 2 versus all other participants (first contrast), *t*(67) = 2.12, *p* = .018 (one-tailed). In turn, the second contrast was not significant, *t*(67) = 1.09, *p* = .279. Follow-up *t*-tests showed that the validity effect (salient cues only) significantly differed from zero only for participants with a single test cue guessing score of 2, Δ_invalid – valid_ = 28 ms, *t*(41) = 3.07, *p* < .002, but not for participants with a guessing score of 1 (Δ_invalid – valid_ = 9 ms, *t*[15] = 0.76, *p* = .462) or 0 (Δ_invalid – valid_ = 12 ms, *t*[11] = 1.19, *p* = .261), respectively.

**Table 2:** Performance in contingency awareness measures, indicated by mean absolute (%) correct answers in Experiments 1 and 2.


EXP	AWARENESS MEASURE	CUE SALIENCY		*t*	DF	*p* (ONE-TAILED)

		SALIENT	NONSALIENT			

1	guessing display following compound cue learning displays (max. correct answers: 8 per cue saliency)	6.0 (75%)	3.9 (49%)	8.09	67	<.001

	guessing display following single cue test displays (max. correct answers per cue saliency: 2)	1.4 (71%)	1.1 (59%)	1.93	67	.029

	post-experimental questions (max. correct answers per cue saliency: 2)	1.6 (82%)	0.7 (34%)	8.72	67	<.001

2	guessing display, following single cue test displays (max. correct answers per cue saliency: 4)	2.8 (71%)	2.0 (50%)	4.81	63	<.001

	post-experimental questions (max. correct answers per cue saliency: 2)	1.1 (53%)	0 (0%)	9.437	63	<.001


*Note*: Exp = Experiment.

For error rates, the MANOVA also revealed a similar trend, although the global interaction missed significance, *F*(2,67) = 1.50, *p* = .231, η_p_^2^ = .04. Again, the part of the interaction that was due to the first contrast was significant, *t*(67) = 1.69, *p* = .047 (one-tailed), whereas the second contrast was not significant, *t*(67) = 0.53, *p* = .593. Follow-up *t*-tests showed that the validity effect for salient cues in error rates significantly differed from zero only for participants with a guessing score of 2, Δ_invalid – valid_ = 6.8%, *t*(41) = 3.68, *p* < .001, but not for participants with a guessing score of 1 (Δ_invalid – valid_ = 3.4%, *t*[15] = 1.62, *p* = .127) or 0 (Δ_invalid – valid_ = 1.3%, *t*[11] = 0.54, *p* = .600), respectively.

In sum, these findings indicate that validity effects for salient cues were obtained only for those participants who became aware of cue-response contingencies.

#### Role of Stimulus Response Bindings in producing selective learning effects

To investigate the role of stimulus response bindings in producing contingency learning effects in salient cues, we ran a multilevel analysis with trial-based predictors as Level 1 variables and participants as Level 2 predictors for trial RT and errors as dependent measures (Table 3), following the example of Giesen and colleagues (2020). For this purpose, every trial was referenced back to the last time the current cue stimulus was presented which could be either a compound learn display or a single cue test display (last previous occurrence), and was coded as having either same or a different response as the current trial (*Previous Response* factor). This allowed us to assess retrieval effects of stimulus-response bindings, with relative advantages when the response of the previous trial repeats (response repetition) in comparison to a situation in which the current trial requires a response that is different from the previous response (response change, cf. Figure 2). Furthermore, we coded the *Distance* between the current trial and the last previous occurrence as a continuous predictor depending on how far the last previous occurrence was (e.g., if the last occurrence was the immediately preceding trial (trial n -1), then Distance was coded as 1; if it was two trials prior to the current trial (trial n - 2), then Distance was coded as 2, etc. After these factors were coded, only the Salient single cue display trials were entered in the analysis as only these trials showed validity effects.

**Table 3:** Multilevel modelling results for both the experiments with reaction time and error data as dependent variable only for salient single cue displays.


	EXPERIMENT 1, REACTION TIME						EXPERIMENT 2, REACTION TIME					
	
	MODEL 1			MODEL 2			MODEL 1			MODEL 2		
			
EFFECTS	*ESTIMATE*	*SE*	*STATISTIC*	*ESTIMATE*	*SE*	*STATISTIC*	*ESTIMATE*	*SE*	*STATISTIC*	*ESTIMATE*	*SE*	*STATISTIC*

Intercept	577.19	9.80	58.89***	577.21	9.80	58.89***	561.41	8.95	62.75***	561.54	8.93	62.87***

V	–8.61	3.14	–2.74*	–6.13	3.50	-1.75	-3.8	3.33	-1.15	–2.95	3.17	-0.93

R				6.89	7.43	0.93				9.34	4.33	2.15*

D				5.13	4.09	1.25				1.24	2.70	0.46

R*D				-8.06	7.94	–1.01				–5.83	5.94	–0.98

**Model Fit**												

**AIC**		30841.632			30866.227		74434.641			74418.470		

	**EXPERIMENT 1, ERROR RATE**						**EXPERIMENT 2, ERROR RATE**					
	
	**MODEL 1**			**MODEL 2**			**MODEL 1**			**MODEL 2**		
			
**EFFECTS**	***ODDS RATIO***	***SE***	***STATISTIC***	***ESTIMATE***	***SE***	***STATISTIC***	***ODDS RATIO***	***SE***	***STATISTIC***	***ESTIMATE***	***SE***	***STATISTIC***

Intercept	0.07	0.01	–22.51***	0.08	0.01	10.66***	0.06	0.01	–21.80***	0.06	0.01	9.57***

V	0.71	0.07	–3.38**	–0.03	0.01	–3.30**	0.75	0.09	–2.32*	–0.01	0	–1.94

R				0	0.01	–0.25				–0.01	0	–1.30

D				0	0.01	–0.36				0	0	–0.42

R*D				–0.05	0.02	–2.77*				0.01	0.01	0.88

**Model Fit**												

**AIC**	1561.057				690.520			2771.990			–1732.221	


*Note*: V: Validity, R: Previous Response, D: Distance from the last occurrence. AIC Akaike’s Information Criterion. * *p* < .05, ** *p* <= .005, *** *p* < .001.

The rationale underlying the multilevel analysis was as follows: In Model 1, only the validity predictor was considered, which should yield evidence for contingency learning (effectively, this would replicate the findings from the aggregated analyses on a trial-based level). In Model 2, we then tested whether the validity effect is the result of episodic SRB retrieval – if this is the case, then the validity effect should no longer be significant as soon as we statistically control for the influence of episodic retrieval (cf. Giesen et al., 2020). In turn, the predictors Previous Response and/or Previous Response by Distance should produce significant effects, which would attest to the impact of episodic retrieval in producing the underlying result pattern.

For RT, Model 1 (which allowed for random intercepts and slopes) included the (contrast-coded) validity factor as (–1 = invalid; 1 = valid) as Level 1 predictor. The results from Model 1 showed a significant effect of validity, β = –8.61, *t* = –2.74, *p* = .006, indicating contingency learning for salient cues (replicating results of follow-up *t*-tests on the aggregated data). For Model 2, Previous Response (2 = response change; 1 = response repetition, mean centered within participant) and Distance (log transformed continuous predictor, mean centered within participant), were added and allowed to interact with each other. Model 2 showed that the effect of validity was not significant anymore after controlling for episodic retrieval of SRBs. However, neither the Previous Response predictor, nor the Previous Response by Distance interaction were significant (see Table 3). This implies that the overall impact of retrieval effects was small and limited, compared with previous studies (Giesen et al., 2020).

While looking at the models with error rate as a dependent variable, controlling for retrieval factors of Previous Response and Distance in Model 2 did not weaken the validity effect, β = –0.03, *t* = –3.30, *p* = .001, even though there was a significant interaction between Previous Response and Distance, β = –0.05, *t* = –2.77, *p* = .006. No other effects were significant (cf. Table 3).

### Discussion

The results from Experiment 1 are noteworthy in several respects: First, participants showed contingency learning as indicated by validity effects in the RT and accuracy data; however, this effect emerged only for salient cues, whereas for nonsalient cues, contingency learning was absent. Hence, the findings show that there is selective learning that occurs in an experimental setup in which two cues compete against each other. Please note that by design, the contingency acquired in the salient cue condition must relate to cue letter identity rather than color, because all cues in test trials were of the same color, which differed from the one used to induce salience. Second, when exploring the role of awareness, we found that contingency awareness played a major role and mediated the effects. That is, learning effects for salient cues only emerged for participants who became aware of the cue-response contingencies (reflected in a guessing score of 2 correct responses), whereas no selective learning emerged for unaware participants (guessing score of 1 or 0, i.e., at or below chance level). These findings largely converge with the data reported by Schmidt and De Houwer (2019). Third, the multilevel analysis revealed that previous response and distance did not mediate the learning effect for salient single cue trials. This suggests that the observed effects of selective contingency learning for salient cues are not due to incidental binding and retrieval processes but arise from the insight/awareness of the cue-response relation.

However, several caveats compromise this conclusion. It is to be noted that the paradigm was not ideal to test for incidental learning. Due to the early appearance of our first on-task contingency awareness measure, one might argue that participants were nudged towards paying more attention to the cues which might have resulted in faster detection of the contingency relation, which was then exploited during the remaining part of the experiment. This is supported by finding that performance in guessing trials after the learning phase was already better for salient than nonsalient cues (Table 2). Hence, although we did not explicitly instruct participants about the underlying contingencies like Schmidt and De Houwer (2019, Exp. 2), we gave participants a strong incentive to optimize their performance by utilizing any hunch or insight into the contingency relations. One could thus argue that these selective learning effects arose from an extrinsic incentive to detect the contingencies and use that knowledge, resulting in selective learning only for salient cues. To rule out this alternative explanation, it has to be tested whether the findings of Experiment 1 replicate in a study in which contingency awareness is only assessed at the very end of the experiment. Under these conditions, guessing trials lose their potential to influence learning by directing participants’ attention to irrelevant cues so that the entire task resembles an incidental learning task.

Second, the paradigm might have rendered it difficult for retrieval effects to exert an influence. The multilevel analysis incorporated last occurrences across intermixed compound cue learning and single cue test displays. However, this might have weakened retrieval effects. That is because the compound cue and single cue displays possibly looked too different from one another, which negatively affected successful retrieval of similar episodes. For instance, consider the case in which the current trial represents a single cue test display, whereas the last occurrence of the same cue represented a compound cue learning display. It might be the case that nothing was retrieved as the stimulus arrangement simply looked very different (different colour; only one letter presented eight times in a circular fashion) compared to the compound cue display which contained eight different letters presented in blue and red. Hence, to increase the likelihood of retrieval effects, test displays should be more similar to learning displays.

## Experiment 2

Experiment 2 was conducted to fix two potential problems of Experiment 1. First, the contingency awareness measure via guessing trials was moved to the very end of the experiment, so that any potential induction of such awareness by the measurement procedure could not impact performance. Second, single cue test displays were made to look more similar to compound cue learning displays. Exact method, hypotheses, exclusion criteria, and planned analyses of Experiment 2 were preregistered on OSF with the AsPredicted.com template (10.17605/OSF.IO/Y29SZ).

### Method

#### Participants

Applying the same criteria as in Experiment 1, 70 new participants (M_age_ = 23.25 years) were recruited. This experiment also was run online via Prolific through hosting it on Pavlovia. The experiment lasted 20-25 minutes and the participants were compensated according to the norms of Prolific (3.30£). This cost estimate was increased by Prolific between the previous experiment and the current experiment, hence the difference in the remuneration costs. Participants also had an opportunity to gain a bonus of 40 pence based on their performance in the guessing trials.

#### Apparatus, stimuli, procedure, and design

Apparatus, stimuli, procedure, and design paralleled Experiment 1 except for the following changes. The main goals of this experiment were to disentangle the role of the on-task contingency awareness measure in producing the effects and to increase the impact of episodic retrieval effects. Thus, the crucial difference between Experiment 1 and 2 lies in the placement of guessing trials and the design of single cue test trial displays. In particular, in contrast to Experiment 1, we no longer used single cue test displays with zero contingency; instead, single cue test displays now also reflected a 9:1 contingency ratio, with 90% valid and 10% invalid trials. Single cue test displays now also looked very similar to compound cue learning trials and always consisted of a salient red cue, a nonsalient blue cue and six random blue distractor letters. Crucially, to test for selective learning effects, half of all single cue test displays contained only the salient cue, but a random blue letter instead of the nonsalient cue; the other half of all single cue test displays contained only the nonsalient cue, but a random red letter instead of the paired salient cue (see Figure 1C).

The next vital change to the paradigm was the placement of contingency guessing display trials, which were moved to the very end of the experiment (see Table 1). Once the entire experiment was complete, 16 contingency guessing trials were presented (8 salient single cue test displays and 8 nonsalient single cue test display followed by a “?” presented centrally instead of the target number). Participants were asked to guess which response is likely and press “e” or “u” based on the type of number they would have expected. Feedback after guessing responses was similar to Experiment 1, as was the funneled debriefing questionnaire which served as an additional measure for subjective contingency awareness.

### Results

#### Data preparation

According to the same criteria as in Experiment 1, three participants were excluded from the analysis due to excessive error rates (>15%). Performance (RT; errors) on target displays served as dependent measure. For RT analyses, 5.55% of all trials were excluded because of incorrect responses; additionally, 5.51% of all trials were outliers or very fast RTs (less than 200ms) and also exempted from analyses. All data and analysis scripts are available on OSF (https://osf.io/sa7eb/).

#### Manipulation checks

First and similar to Experiment 1, the contingency effect for the compound cue display learning trials was analyzed. At the level of RT, the *t*-test yielded a significant effect of contingency learning for target displays following valid versus invalid compound cue learning displays, *t*(66) = 1.84 (one-tailed), *p* = .035, d_z_ = 0.22, with a mean difference of Δ_invalid – valid_ = 11 ms. The error rate also showed a significant difference between the performance for target displays following valid versus invalid learning trials, *t*(66) = 3.68, *p* < .001, d_z_ = 0.44, with a mean difference of Δ_invalid – valid_ = 5.6% errors.

Second, the manipulation check for saliency (comparing those trials in which the target digit appeared at the position of the salient vs. non-salient cue) was analyzed. Participants performed 12 ms faster when the target was presented at the position of the salient compared with the nonsalient cue, however, this difference failed the significance criterion, *t*(66) = 1.57, *p* = .060 (one-tailed). Similarly, there was no significant cuing effect of the salient position in the error data, *t*(66) = 0.29, *p* = .387.^2^ Follow-up analyses suggested that rather than rendering the manipulation unsuccessful, these findings possibly reflect low reliability due to using only few manipulation check trials (for details, see footnote 2).

#### Contingency learning effects

To test for selective contingency learning effects, performance on target displays following single cue test displays were analyzed. A 2 (Cue Saliency: salient vs. nonsalient) × 2 (Validity: valid vs. invalid) repeated-measures ANOVA was computed on the aggregated means (RT; errors). For RTs, the interaction between validity and saliency just missed significance, *F*(1,66) = 3.29, *p* = .074, η_p_^2^ = .05. Given the directional nature of our hypothesis, we compared contingency learning effects for salient versus nonsalient single cues in a one-tailed paired *t*-test, which was significant *t*(66) = –1.81, *p* = .037, d_z_ = .0.22, indicating that contingency learning effects were significantly larger for salient than for nonsalient cues. Follow-up *t*-tests showed that the contingency learning effect was not significantly different from zero neither for the salient cues, *t*(66) = 1.37, *p* = .088, Δ_invalid – valid_ = 10.02 ms, d_z_ = 0.17, nor for nonsalient cues *t*(66) = –1.19, *p* = .881, Δ_invalid – valid_ = –5.83 ms, d_z_ = 0.14. No other effect was significant (*F* < 1, *p* > .5).

**Figure 4:**
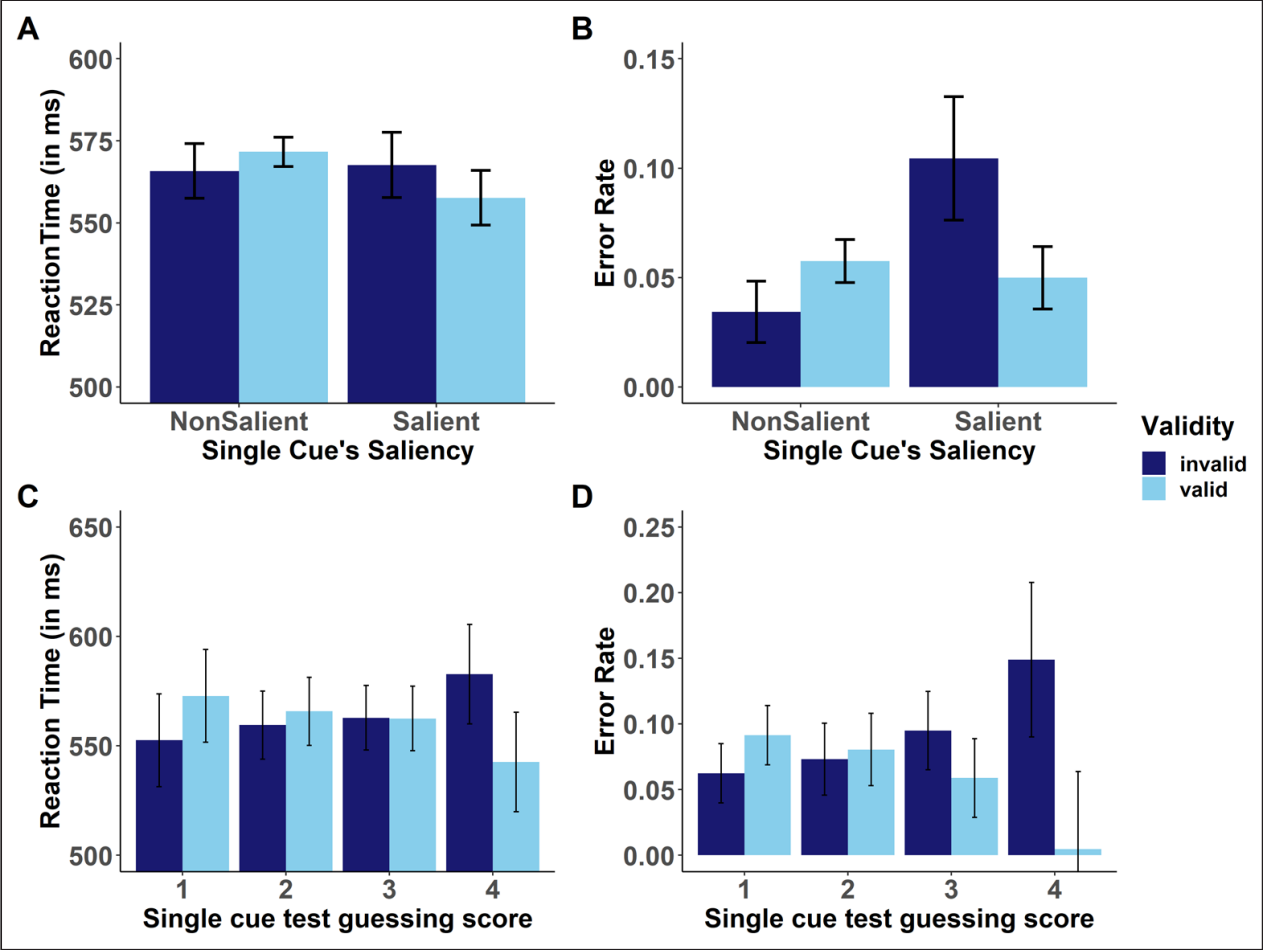
Results of Experiment 2. Top row: Performance for valid and invalid target display trials per saliency of the single cue display that preceded the target for mean RT (A) and mean error rates (B). Bottom row: Validity effects for salient cues as a function of participants’ achieved guessing score following single cue test displays for mean RT (C) and mean error rates (D). Error bars represent 95% CI from standard error of each condition as explained in Morey (2008).

For error rates, however, the interaction between saliency and validity was significant, *F*(1,66) = 14.7, *p* < .001, η_p_^2^ = 0.18. Follow-up one-tailed *t*-tests showed that as expected, the contingency learning effect was significantly different from zero for the salient cues, *t*(66) = 3.03, *p* = .001, Δ_invalid – valid_ = 5.5%, d_z_ = 0.37, but was not significant for nonsalient cues *t*(66) = -3.06, *p* = .99, Δ_invalid – valid_ = -2.3%, d_z_ = 0.37 (see Figure 4). Together, results of Experiment 2 replicate findings from Experiment 1: Contingency learning effects were stronger following salient single cue test displays than following nonsalient single cue test displays (descriptively, the latter showed reversed effects). This implies that contingencies of salient, but not the nonsalient cues were learnt during compound learning displays.

#### Role of contingency awareness

To investigate the role of contingency awareness, we again analyzed the answers in the guessing trials presented at the end of the experiment as a function of cue saliency. Similar to Experiment 1, performance was significantly higher for salient compared with nonsalient cues (Table 2) in Experiment 2, too. We then analyzed validity effects for salient cues as a function of participants’ mean accuracy scores in single cue guessing trials, which varied between 1 and 4 in Experiment 2. Overall, N = 10 participants had an accuracy score of 1 and N = 15 participants had an accuracy score of 2, these were regarded as participants who performed at chance level. Additionally, N = 18 participants had an accuracy score of 3, and N = 24 had an accuracy score of 4; these participants were regarded as participants who performed above chance level. For RT, a 2 (validity) × 4 (single cue contingency awareness score) MANOVA with three orthogonal contrasts on the last factor was computed (contrast 1 compared performance of participants who performed below vs. above chance level [–1 –1 1 1]; contrast 2 compared participants who performed below chance level [–1 1 0 0]; contrast 3 compared participants who performed better than chance level [0 0 –1 1]). This MANOVA yielded a significant interaction, *F*(3,63) = 3.83, *p* = .014, η_p_^2^ = 0.15. Planned contrasts showed that this interaction was due to the first contrast, *t*(63) = 2.31, *p* = .024 (reflecting stronger validity effects for participants who performed better than chance), as well as the third contrast, *t*(63) = 2.25, *p* = .027 (reflecting stronger validity effects for participants with a guessing score of 4 vs. 3). The second contrast was not significant, *t*(63) = 0.61, *p* = .547 (indicating that validity effects did not differ for participants with a guessing score of 1 vs. 2). Follow-up *t*-tests showed that the validity effect for salient cues was significantly different from zero only for participants with a guessing score of 4 (Δ_invalid – valid_ = 40 ms, *t*(23) = 2.58, *p* = .017), but was absent for all other participants (guessing score 3: Δ_invalid – valid_ = 0 ms, |*t*| < 1, *p* = .974; guessing score 2: Δ_invalid – valid_ = –6 ms, |*t*| < 1, *p* = .549; guessing score 1: Δ_invalid – valid_ = –20 ms, *t*(9) = 1.53, *p* = .16; see Figure 4).

For error rates, the same effect pattern emerged, as the global interaction was significant, *F*(3,63) = 6.20, *p* = .001, η_p_^2^ = 0.23. Planned contrasts showed that this interaction was due to the first contrast, *t*(63) = 3.18, *p* = .002, as well as the third contrast, *t*(63) = 2.61, *p* = .011. The second contrast was not significant, *t*(63) = 0.40, *p* = .690. Follow-up *t*-tests showed that the validity effect for salient cues was significantly different from zero only for participants with a guessing score of 4 (Δ_invalid – valid_ = 14.4%, *t*(23) = 3.57, *p* = .002), but was absent for all other participants (guessing score 3: Δ_invalid – valid_ = 3.6%, *t*(17) = 1.80, *p* = .089; guessing score 2: Δ_invalid – valid_ = –0.7%, |*t*| < 1, *p* = .692; guessing score 1: Δ_invalid – valid_ = –2.9%, *t*(9) = 2.06, *p* = .07; see Figure 6). In sum, these findings indicate that awareness mediated contingency learning effects for salient cues in Experiment 2, too.

#### Role of Stimulus Response Bindings in producing selective learning effects

Similar to the analysis conducted for Experiment 1, we used multilevel modelling to test the influence of retrieval of SRB in producing selective learning effects with only the salient single cue trials. With Reaction Time as a dependent variable, Model 1 was computed using only validity (contrast-coded) as a level 1 predictor which showed that the learning effect was not significant for salient cues, β = –3.83, *t* = –1.15, *p* = .250 (replicating findings from the follow up *t*-tests on the aggregated data). When the factors related to retrieval of transient bindings, namely Previous Response (2 = Response Change, 1 = Response Repetition, mean centered within participant) and Distance (log transformed continuous predictor, mean centered within participant) were added as level 1 predictors allowing for interactions between Previous Response and Distance in Model 2, there was a significant effect of Previous Response, β = –9.34, *t* = 2.15, *p* = .031 indicating that the retrieval of SRB contributed to the performance and explained variance above and beyond the validity factor. However, since there was no significant effect of validity to begin with, this retrieval effect is not responsible for performance on salient single cue displays. No other effect was significant (see Table 3).

When running a similar multilevel analysis on error rates, Model 1 yielded a significant effect of validity, β = 0.75, *t =* –2.32, *p* = .010 (one-tailed). In Model 2, the results showed that the validity effect became slightly weaker, β = –0.01, *t* = –1.94, *p* = .025 (one-tailed), but was still significant, after controlling for episodic retrieval effects; however, the effects of Previous Response, Distance, and their interaction were not significant. Therefore, these results show that retrieval of SRB does not explain the selective learning for salient cues.

### Discussion

As in Experiment 1, we obtained a selective contingency learning effect in Experiment 2, too. That is, contingency learning, as indicated by validity effects in the RT and accuracy data, was stronger for salient cues than for nonsalient cues. Hence, the findings of Experiment 2 nicely replicate the findings from Experiment 1. Importantly, due to the procedural changes in Experiment 2, we can exclude that the on-task measure of contingency awareness was responsible in producing these effects because contingency awareness was only assessed at the very end of the study.

Although it is safe to say that these effects were not driven by the incentive to learn by the on-task awareness measure, data from Experiment 2 clearly show that awareness still played a central role. Selective learning effects were only obtained for participants who exhibited awareness of the underlying contingencies over the course of the study (reflected in a contingency awareness score of four correct answers – out of four – assessed at the very end of the experiment). Against this background, it comes as no surprise that, similar to Experiment 1, the multilevel analysis yielded no support for a mediating role of retrieval of transient SRB’s in Experiment 2, either. Importantly, this pattern emerged although the conditions were more beneficial for episodic retrieval processes, since single test displays were now more similar to compound learning displays and detection of contingencies was more difficult, given that the on-task measure was postponed to the end of the study.

In sum, the obtained results support the view that selective contingency learning effects in our study emerged only for those participants who were later able to verbalize the underlying contingency relations, rather than results of incidental binding and retrieval processes. This means that under specific conditions, participants will consciously pick up and learn cue-response contingency relations and later on apply their knowledge of the detected rules. Such an application of detected contingency relations renders episodic retrieval processes unnecessary. Moreover, contingencies between more salient cues and responses have a much higher probability to be detected and acted upon than contingencies between nonsalient cues and responses. Our findings therefore nicely complement but also extend the results from Schmidt and De Houwer (2019) by showing that even without explicitly instructing participants to notice the contingencies, there is a preference for learning a salient cue over a nonsalient cue in a contingency learning task as the consequence of detecting and having awareness of the underlying contingency relations and utilizing them for later performance.

## General Discussion

The aims of the present study were two-fold: First, we aimed to demonstrate selective contingency learning in an experimental setup where two competing cues differ in saliency. Second, we aimed to find out more about the underlying factors that produced these learning effects. In particular, we aimed to elucidate the role of contingency awareness and retrieval of incidental stimulus-response bindings in producing learning effects for salient cue.

In two pre-registered experiments, saliency of irrelevant cues that were highly predictive of subsequent responses to target numbers was manipulated experimentally by visually highlighting one cue (rendering it salient), whereas the other cue was simply colored as six other random letters (rendering it nonsalient). Manipulation checks showed that this saliency manipulation was successful (cf. footnote 2), as target detection on manipulation check displays was faster for targets that appeared at the location of the salient versus nonsalient cue. Intriguingly, in compound cue learning displays, both salient and nonsalient cues were equally predictive of upcoming target responses. However, when tested in isolation in single cue test displays, participants showed contingency learning effects only for salient cues. In turn, contingency learning effects were not only smaller, but virtually absent for nonsalient cues. The results show a difference in the magnitude of learning for the salient and nonsalient predictive cue, that is, they show selectivity in learning, indicating that learning is not the same for all kinds of predictive stimuli. Hence, our study is the first that documents selective contingency learning in an experimental task where two task-irrelevant cues of similar modality and only differing in relative saliency compete against each other during learning.

With regard to our second research aim, analyses revealed that selective learning effects were mediated by level of contingency awareness. In particular, the selective learning effects only emerged for participants who become aware of the contingency relations (and the predictive nature of salient cues in particular).

One may argue that larger validity effects for salient as compared to nonsalient cues reflect the generally higher potential of salient cues to be noticed while picking up contingencies between cues and responses, irrespective of whether another cue, and thus cue competition, was possible or not. So, a more fine-grained analysis of cue competition might employ a control learning condition with only one of the cues being present and thus no competition. If such a condition would indeed show learning effects even for nonsalient cues, this would also allow for an investigation of phenomena such as overshadowing in contingency learning (cf. footnote 1). Yet, the present observation that awareness mediated contingency learning in the present paradigm suggests that any such possible difference between competing versus non-competing cues is likely mediated by awareness of cue-response contingencies. In other words, saliency of cues, be another cue be present or not, is probably not the driving force behind contingency learning, rather it is the awareness of contingencies which is fostered by saliency that drives the selective learning process for only salient cues. In this regard, it would be interesting to explore whether overshadowing effects might also go along with differences in contingency awareness (that is, whether being aware of a cue-response contingency reduces learning about another cue-response contingency).

Another finding of high relevance for our second research aim are the results of the multilevel analyses, which were modelled after Giesen and colleagues (2020) to test whether retrieval of short-lived stimulus-response binding and retrieval can explain the observed learning effects. Results of the multilevel analyses support the view that this was not the case. In both experiments, statistically controlling for the influence of episodic retrieval processes in Model 2 did reduce the impact of the validity predictor, but failed to produce significant findings for retrieval related predictors to incrementally account for variance not already explained by the validity predictor. One could argue that the conditions of Experiment 1 were not ideal for episodic retrieval processes to be successful (since learning and test displays looked rather different from each other), which might account for the low impact of episodic retrieval in that study. However, the result was not changed in Experiment 2, although single test displays were more similar to learning displays both in terms of visual appearance and also in terms of contingency ratio. When evaluating these null findings, it should be taken into consideration that the displays of the test trial and of the last occurrence episode still differed strongly in their appearance. Most importantly, the spatial position of the cue typically differed between the current trial and the last occurrence, and the composition of the letter displays was also different (only 1 or 2 in case of compound cue letter repeated, while remaining letters were different each time). In spite of the displays sharing more resemblance in the second experiment, these other crucial differences and might have weakened/prevented an influence of retrieval on learning the salient cues. Together, the findings of both experiments support the conclusion that the learning effect for salient cues is mediated by a conscious detection of the underlying contingencies, which renders the influence of episodic retrieval processes of accumulated SRB as negligible.

At this point we want to highlight that effects of the most recent learning episode per se do not provide unambiguous evidence for an influence of episodic binding and retrieval processes. In principle, effects reflecting the influence of a single (most recent) episode can also influence results via more cumulative, abstractionist processes of knowledge acquisition (e.g., association formation). If, however, effects of contingency learning (CL) are completely eliminated after controlling for just one (the most recent) episode, as it was shown in previous analyses of contingency learning (Giesen et al., 2020; Schmidt et al., 2020; see also Rothermund et al., 2022) then the findings are no longer amenable to an explanation in terms of abstractionist contingency learning. Contingency learning (or association formation) by definition requires multiple episodes to explain the results because the very concept of a contingency implies repeated encounters of stimulus (or response) combinations. Relatedly, a core feature of abstractionist learning is that enduring representations (“associations”) are formed that represent the accumulated knowledge and integrated experience that has been gathered across multiple learning episodes. Although recency may increase the impact of the last episode over other episodes (e.g., the learning algorithm may assign a larger weight to the current compared to previous episodes; cf. Barsalou, 1990), it would be a conceptual stretch to assume that associations can be formed, and also that already existing associations can be completely reversed, by a single event (this would imply assigning a weight of one to the current episode and weights of zero to all previous events). In such an extreme case, basic conceptual requirements of concepts like contingency or knowledge accumulation that are implied in an abstractionist conception of learning are violated. This pattern, however, is exactly what has been shown in previous studies (Giesen et al., 2020; Schmidt et al., 2020; Rothermund et al., 2022). We took a similar perspective in our study, in which we tested whether effects of contingency learning can be completely eliminated by controlling for the effects of the most recent learning episode. This was not the case in our study, so we rejected the hypothesis that CL effects were mediated by episodic retrieval. If such a pattern of complete mediation and elimination of CL effects by just one episode had occurred, however, then we would have felt justified to reject abstractionist explanations of this pattern of results.

### Limitations

We want to point out that the kind of contingency effects which were investigated in the present study are all but trivial: First, cues were task-irrelevant; second, two irrelevant cues compete against each other. Third, cues appear on a different display than target stimuli, which makes it harder for participants to detect co-occurrences and contingencies. For instance, Carlson and Flowers (1996) showed that participants unintentionally use contingencies when these exist between target and distractors presented in the very same trial. However, when distractors on trial n–1 predicted targets on trial n, unintentional use of contingencies was absent and only participants who were explicitly informed made use of contingencies and came up with intentional prediction strategies. On a more general level, this could imply that in the color-word contingency learning paradigm as endorsed by Giesen and colleagues (2020; see also Schmidt et al., 2020) unintentional detection and use of contingencies is more likely, since relevant (color) and irrelevant (word meaning) features are presented in the very same trial display. Similarly, it is plausible that emergence and retrieval of transient SRB increases in a setup in which relevant and irrelevant stimulus features belong to the same object (i.e., a word; cf. Frings & Rothermund, 2011) than when both are separated in time, space, and object-belongingness due to being presented in different displays. However, if task-relevant targets and irrelevant cues are presented on the same trial, processing of irrelevant cues might suffer on the whole, especially with the complex stimulus arrangement of the current study. Adding to these concerns, our study employed a technique in which displays that comprised of eight different stimuli were shown in order to efficiently manipulate the saliency of the cues. This manipulation, however, comes with the cost that retrieval effects are rendered unlikely due to the small overlap between current and previous episodes. Even our second experiment might thus not have provided optimal conditions for testing the influence of automatic retrieval on selective contingency learning. In sum then, finding a technique that allows for (a) a simultaneous presentation of cues, targets, and responses, and (b) also allows for an efficient manipulation of saliency with only two cues might be needed in order to more validly evaluate the influence of retrieval on contingency learning.

### Theoretical implications

The present findings are of theoretical importance in several respects. First, there is an ongoing debate on whether SRBs are relevant for longer lasting learning effects. Results in this regard are still few and mixed. Some studies do find evidence that SRB effects show a strong resemblance to learning phenomena and can fully account for contingency learning effects (Giesen et al., 2020; Schmidt et al., 2020). Other studies, in turn, do not support this unitary view, but rather argue for a dissociation of short-lived binding and more permanent learning phenomena (Colzato et al., 2006; Herwig & Waszak, 2012; Moeller & Frings, 2014). In this respect, our data do not support the view that accumulated bindings can account for selective contingency learning effects, and thus rather support the view that SRB effects dissociate from learning effects. However, this conclusion might be premature, at least as far as more complex learning phenomena are concerned. In other words, it could be that the chosen trial sequence gave more weight to strategic processes like conscious rule detection, which diminished the role of episodic retrieval processes (cf. Limitations).

Our findings are also relevant for ongoing research on binding and retrieval processes. For instance, according to the recent *Binding and Retrieval in Action Control* framework, (Frings et al., 2020) binding and retrieval processes are considered as being independent. Therefore, both should in principle be open to modulations by bottom-up factors like stimulus saliency. Recent findings by Schmalbrock and colleagues (2021) show that stimulus-response binding is stronger for salient targets. In this respect, one could understand the multi-level analyses of the present study as a test for a modulation of retrieval processes by saliency, with the assumption that salient stimuli grab more attention and are thus more likely to retrieve previously established SRB’s from memory. If that were the case, we should have observed significant interactions between Previous Response and Distance factors for salient trials which reflect SRB effects in the multi-level analyses. However, none of these effects emerged in our study. As mentioned above, the task design might have been detrimental for the emergence of strong cue-based binding and retrieval effects (cf. Limitations).

Nevertheless, it is possible that in relation to more complex learning processes involving multiple cues, the role of awareness supersedes the role of episodic retrieval in contributing to the selective learning effects for salient cues which was illustrated in the findings of our experiments. Future studies should aim to further explore the relationship between episodic binding and retrieval effects and other learning processes and investigate the extent to which the former can explain the latter. All in all, our study provides an outlook in this direction of dissociating the role of insight and transient stimulus response bindings in contingency learning of cues that differ in saliency.

## Data Accessibility Statement

All the materials related to experiment including data and the R scripts utilized for data analyses are made publicly available through the OSF Framework (https://osf.io/sa7eb/).

## Footnotes

Our initial goal (cf. preregistrations) was aimed at exploring overshadowing in contingency learning. However, our experimental design did not allow for a control condition that assesses learning when salient and nonsalient cues are presented in isolation, which limits possible inferences regarding overshadowing. Thus, while the present experiments speak to the question of how saliency shapes learning preferences, they cannot directly address whether a salient cue can overshadow or reduce the learning effect for a nonsalient cue.

A pooled analysis on saliency manipulation check trials across the two experiments revealed a significant effect of saliency that was equally effective in both Experiments: A 2 (target position: salient vs. nonsalient cue) × 2 (Exp: 1 vs. 2) ANOVA yielded a main effect of target position, *F*(1,35) = 7.97, *p* = .005 that shows faster performance when target is at the salient cue’s position compared to the nonsalient cue. There was neither an effect of Experiment, nor an interaction between Target Position and the Experiment (all *Fs* < 1, *p*s > .5). We thus feel justified to assume that the saliency manipulation also worked properly in Experiment 2.
